# Supplementary material for: Iterative improvement in the automatic modular design of robot swarms
Source: PeerJ Comput Sci. 2020 Dec 7;6:e322. doi: 10.7717/peerj-cs.322 (PMC7924708; doi:10.7717/peerj-cs.322)
Supplement: Supplemental Information 3 [file peerj-cs-06-322-s003.zip › argos3/doc/api/standalone/a00410_source.html]

ARGoS: core/wrappers/lua/lua\_utility.cpp Source File


- Main Page
- Related Pages
- Namespaces
- Classes
- Files

- File List
- File Members

# core/wrappers/lua/lua\_utility.cpp

Go to the documentation of this file.

```
00001 
00007 #include "lua_utility.h"
00008 #include <argos3/core/utility/configuration/argos_exception.h>
00009 #include <argos3/core/utility/math/angles.h>
00010 #include <argos3/core/utility/math/vector2.h>
00011 #include <argos3/core/utility/math/vector3.h>
00012 #include <argos3/core/utility/math/quaternion.h>
00013 #include <argos3/core/utility/datatypes/color.h>
00014 
00015 namespace argos {
00016 
00017    /****************************************/
00018    /****************************************/
00019 
00020    static const CRange<Real> UNIT(0.0f, 1.0f);
00021 
00022    int LuaRNGBernoulli(lua_State* pt_state) {
00023       /* Check number of parameters */
00024       if(lua_gettop(pt_state) > 1) {
00025          return luaL_error(pt_state, "robot.random.bernoulli() expects 0 or 1 arguments");
00026       }
00027       /* Get RNG instance */
00028       CRandom::CRNG* pcRNG = CLuaUtility::GetDeviceInstance<CRandom::CRNG>(pt_state, "random");
00029       /* Perform wanted action */
00030       if(lua_gettop(pt_state) == 0) {
00031          /* Return random number */
00032          lua_pushnumber(pt_state, pcRNG->Bernoulli());
00033          return 1;
00034       }
00035       else {
00036          /* Check parameter */
00037          luaL_checktype(pt_state, 1, LUA_TNUMBER);
00038          /* Return random number */
00039          lua_pushnumber(pt_state,
00040                         pcRNG->Bernoulli(lua_tonumber(pt_state, 1)));
00041          return 1;
00042       }
00043       /* Can't reach this point */
00044       return 0;
00045    }
00046 
00047    int LuaRNGUniform(lua_State* pt_state) {
00048       /* Check number of parameters */
00049       if(lua_gettop(pt_state) > 2) {
00050          return luaL_error(pt_state, "robot.random.uniform() expects 0, 1, or 2 arguments");
00051       }
00052       /* Get RNG instance */
00053       CRandom::CRNG* pcRNG = CLuaUtility::GetDeviceInstance<CRandom::CRNG>(pt_state, "random");
00054       /* Perform wanted action */
00055       if(lua_gettop(pt_state) == 0) {
00056          /* Return a number between 0 and 1 */
00057          lua_pushnumber(pt_state, pcRNG->Uniform(UNIT));
00058          return 1;
00059       }
00060       else if(lua_gettop(pt_state) == 1) {
00061          /* Check parameter */
00062          luaL_checktype(pt_state, 1, LUA_TNUMBER);
00063          /* Return a number between 0 and the max */
00064          lua_pushnumber(pt_state,
00065                         pcRNG->Uniform(CRange<Real>(0,
00066                                                     lua_tonumber(pt_state, 1))));
00067          return 1;
00068       }
00069       else {
00070          /* Check parameters */
00071          luaL_checktype(pt_state, 1, LUA_TNUMBER);
00072          luaL_checktype(pt_state, 2, LUA_TNUMBER);
00073          /* Return a number between min and max */
00074          lua_pushnumber(pt_state,
00075                         pcRNG->Uniform(CRange<Real>(lua_tonumber(pt_state, 1),
00076                                                     lua_tonumber(pt_state, 2))));
00077          return 1;
00078       }
00079       /* Can't reach this point */
00080       return 0;
00081    }
00082 
00083    int LuaRNGUniformInt(lua_State* pt_state) {
00084       /* Check number of parameters */
00085       if(lua_gettop(pt_state) > 2) {
00086          return luaL_error(pt_state, "robot.random.uniform_int() expects 1 or 2 arguments");
00087       }
00088       /* Get RNG instance */
00089       CRandom::CRNG* pcRNG = CLuaUtility::GetDeviceInstance<CRandom::CRNG>(pt_state, "random");
00090       /* Perform wanted action */
00091       if(lua_gettop(pt_state) == 1) {
00092          /* Check parameter */
00093          luaL_checktype(pt_state, 1, LUA_TNUMBER);
00094          /* Return a number between 0 and the max */
00095          lua_pushnumber(pt_state,
00096                         pcRNG->Uniform(CRange<UInt32>(0,
00097                                                       Floor(lua_tonumber(pt_state, 1)))));
00098          return 1;
00099       }
00100       else {
00101          /* Check parameters */
00102          luaL_checktype(pt_state, 1, LUA_TNUMBER);
00103          luaL_checktype(pt_state, 2, LUA_TNUMBER);
00104          /* Return a number between min and max */
00105          lua_pushnumber(pt_state,
00106                         pcRNG->Uniform(CRange<SInt32>(Floor(lua_tonumber(pt_state, 1)),
00107                                                       Floor(lua_tonumber(pt_state, 2)))));
00108          return 1;
00109       }
00110       /* Can't reach this point */
00111       return 0;
00112    }
00113 
00114    int LuaRNGExponential(lua_State* pt_state) {
00115       /* Check number of parameters */
00116       if(lua_gettop(pt_state) != 1) {
00117          return luaL_error(pt_state, "robot.random.exponential() expects 1 argument");
00118       }
00119       /* Get RNG instance */
00120       CRandom::CRNG* pcRNG = CLuaUtility::GetDeviceInstance<CRandom::CRNG>(pt_state, "random");
00121       /* Check parameter */
00122       luaL_checktype(pt_state, 1, LUA_TNUMBER);
00123       /* Return random number */
00124       lua_pushnumber(pt_state,
00125                      pcRNG->Exponential(lua_tonumber(pt_state, 1)));
00126       return 1;
00127    }
00128 
00129    int LuaRNGGaussian(lua_State* pt_state) {
00130       /* Check number of parameters */
00131       if(lua_gettop(pt_state) != 1 && lua_gettop(pt_state) != 2) {
00132          return luaL_error(pt_state, "robot.random.gaussian() expects 1 or 2 arguments");
00133       }
00134       /* Get RNG instance */
00135       CRandom::CRNG* pcRNG = CLuaUtility::GetDeviceInstance<CRandom::CRNG>(pt_state, "random");
00136       /* Perform wanted action */
00137       if(lua_gettop(pt_state) == 1) {
00138          /* Check parameter */
00139          luaL_checktype(pt_state, 1, LUA_TNUMBER);
00140          /* Return random number */
00141          lua_pushnumber(pt_state, pcRNG->Gaussian(lua_tonumber(pt_state, 1)));
00142          return 1;
00143       }
00144       else {
00145          /* Check parameters */
00146          luaL_checktype(pt_state, 1, LUA_TNUMBER);
00147          luaL_checktype(pt_state, 2, LUA_TNUMBER);
00148          /* Return random number */
00149          lua_pushnumber(pt_state,
00150                         pcRNG->Gaussian(lua_tonumber(pt_state, 1),
00151                                         lua_tonumber(pt_state, 2)));
00152          return 1;
00153       }
00154       /* Can't reach this point */
00155       return 0;
00156    }
00157 
00158    /****************************************/
00159    /****************************************/
00160 
00161    bool CLuaUtility::LoadScript(lua_State* pt_state,
00162                                 const std::string& str_filename) {
00163       if(luaL_loadfile(pt_state, str_filename.c_str())) {
00164          return false;
00165       }
00166       if(lua_pcall(pt_state, 0, 0, 0)) {
00167          return false;
00168       }
00169       return true;
00170    }
00171 
00172    /****************************************/
00173    /****************************************/
00174 
00175    bool CLuaUtility::CallLuaFunction(lua_State* pt_state,
00176                                      const std::string& str_function) {
00177       lua_getglobal(pt_state, str_function.c_str());
00178       if(lua_pcall(pt_state, 0, 0, 0)) {
00179          return false;
00180       }
00181       return true;
00182    }
00183 
00184    /****************************************/
00185    /****************************************/
00186 
00187    void PrintStackEntry(CARGoSLog& c_log, lua_State* pt_state, SInt32 n_index) {
00188       switch(lua_type(pt_state, n_index)) {
00189          case LUA_TBOOLEAN: c_log << lua_toboolean(pt_state, n_index); break;
00190          case LUA_TNUMBER: c_log << lua_tonumber(pt_state, n_index); break;
00191          case LUA_TSTRING: c_log << lua_tostring(pt_state, n_index); break;
00192          default: c_log << lua_topointer(pt_state, n_index); break;
00193       }
00194    }
00195 
00196    void RecursivePrintGlobals(CARGoSLog& c_log,
00197                               lua_State* pt_state,
00198                               size_t un_depth) {
00199       for(size_t i = 0; i < un_depth; ++i) {
00200          c_log << " ";
00201       }
00202       PrintStackEntry(c_log, pt_state, -2);
00203       c_log << " [" << lua_typename(pt_state, lua_type(pt_state, -1)) << "] ";
00204       if(lua_istable(pt_state, -1)) {
00205          c_log << std::endl;
00206          lua_pushnil(pt_state);
00207          while(lua_next(pt_state, -2)) {
00208             if(lua_type(pt_state, -1) != LUA_TFUNCTION &&
00209                (lua_type(pt_state, -2) != LUA_TSTRING ||
00210                 (std::string(lua_tostring(pt_state, -2)) != "_G" &&
00211                  std::string(lua_tostring(pt_state, -2)) != "_VERSION" &&
00212                  std::string(lua_tostring(pt_state, -2)) != "package" &&
00213                  std::string(lua_tostring(pt_state, -2)) != "string" &&
00214                  std::string(lua_tostring(pt_state, -2)) != "os" &&
00215                  std::string(lua_tostring(pt_state, -2)) != "io" &&
00216                  std::string(lua_tostring(pt_state, -2)) != "math" &&
00217                  std::string(lua_tostring(pt_state, -2)) != "debug" &&
00218                  std::string(lua_tostring(pt_state, -2)) != "coroutine" &&
00219                  std::string(lua_tostring(pt_state, -2)) != "table"))) {
00220                RecursivePrintGlobals(c_log, pt_state, un_depth+1);
00221             }
00222             lua_pop(pt_state, 1);
00223          }
00224       }
00225       else {
00226          PrintStackEntry(c_log, pt_state, -1);
00227          c_log << std::endl;
00228       }
00229    }
00230 
00231    void CLuaUtility::PrintGlobals(CARGoSLog& c_log,
00232                                   lua_State* pt_state) {
00233       c_log << "*** LUA GLOBALS START ***" << std::endl;
00234       lua_getglobal(pt_state, "_G");
00235       RecursivePrintGlobals(c_log, pt_state, 0);
00236       lua_pop(pt_state, 1);
00237       c_log << "*** LUA GLOBALS END ***" << std::endl;
00238 #ifdef ARGOS_THREADSAFE_LOG
00239       c_log.Flush();
00240 #endif
00241    }
00242 
00243    /****************************************/
00244    /****************************************/
00245 
00246    void CLuaUtility::PrintStack(CARGoSLog& c_log,
00247                                 lua_State* pt_state) {
00248       c_log << "*** LUA STACK START ***" << std::endl;
00249       size_t unTop = lua_gettop(pt_state);
00250       c_log << "Elements in stack: " << unTop << std::endl;
00251       for(size_t i = unTop; i > 0; --i) {
00252          c_log << "#" << i << " [" << lua_typename(pt_state, lua_type(pt_state, i)) << "] ";
00253          PrintStackEntry(c_log, pt_state, i);
00254          c_log << std::endl;
00255       }
00256       c_log << "*** LUA STACK END ***" << std::endl;
00257 #ifdef ARGOS_THREADSAFE_LOG
00258       c_log.Flush();
00259 #endif
00260    }
00261 
00262    /****************************************/
00263    /****************************************/
00264 
00265    void CLuaUtility::RegisterLoggerWrapper(lua_State* pt_state) {
00266       lua_register(pt_state, "log", LOGWrapper);
00267       lua_register(pt_state, "logerr", LOGERRWrapper);
00268    }
00269    
00270    /****************************************/
00271    /****************************************/
00272 
00273    void CLuaUtility::RegisterRNG(lua_State* pt_state,
00274                                  CRandom::CRNG* pc_rng) {
00275       pc_rng->Reset();
00276       OpenRobotStateTable(pt_state, "random");
00277       AddToTable(pt_state, "_instance", pc_rng);
00278       AddToTable(pt_state, "bernoulli", &LuaRNGBernoulli);
00279       AddToTable(pt_state, "uniform", &LuaRNGUniform);
00280       AddToTable(pt_state, "uniform_int", &LuaRNGUniformInt);
00281       AddToTable(pt_state, "exponential", &LuaRNGExponential);
00282       AddToTable(pt_state, "gaussian", &LuaRNGGaussian);
00283       CloseRobotStateTable(pt_state);
00284    }
00285 
00286    /****************************************/
00287    /****************************************/
00288 
00289    void CLuaUtility::OpenRobotStateTable(lua_State* pt_state,
00290                                          const std::string& str_key) {
00291       lua_pushstring(pt_state, str_key.c_str());
00292       lua_rawget(pt_state, -2);
00293       if(lua_isnil(pt_state, -1)) {
00294          lua_pop(pt_state, 1);
00295          StartTable(pt_state, str_key);
00296          EndTable(pt_state);
00297          lua_pushstring(pt_state, str_key.c_str());
00298          lua_rawget(pt_state, -2);
00299       }
00300    }
00301 
00302    /****************************************/
00303    /****************************************/
00304 
00305    void CLuaUtility::CloseRobotStateTable(lua_State* pt_state) {
00306       lua_pop(pt_state, 1);
00307    }
00308 
00309    /****************************************/
00310    /****************************************/
00311 
00312    void CLuaUtility::StartTable(lua_State* pt_state,
00313                                 const std::string& str_key) {
00314       lua_pushstring(pt_state, str_key.c_str());
00315       lua_newtable (pt_state);
00316    }
00317 
00318    /****************************************/
00319    /****************************************/
00320 
00321    void CLuaUtility::StartTable(lua_State* pt_state,
00322                                 int n_key) {
00323       lua_pushnumber(pt_state, n_key);
00324       lua_newtable  (pt_state);
00325    }
00326 
00327    /****************************************/
00328    /****************************************/
00329 
00330    void CLuaUtility::EndTable(lua_State* pt_state) {
00331       lua_settable(pt_state, -3);
00332    }
00333    
00334    /****************************************/
00335    /****************************************/
00336 
00337    void CLuaUtility::AddToTable(lua_State* pt_state,
00338                                 const std::string& str_key,
00339                                 void* pt_data) {
00340       lua_pushstring       (pt_state, str_key.c_str());
00341       lua_pushlightuserdata(pt_state, pt_data        );
00342       lua_settable         (pt_state, -3             );
00343    }
00344 
00345    /****************************************/
00346    /****************************************/
00347 
00348    void CLuaUtility::AddToTable(lua_State* pt_state,
00349                                 const std::string& str_key,
00350                                 lua_CFunction t_data) {
00351       lua_pushstring   (pt_state, str_key.c_str());
00352       lua_pushcfunction(pt_state, t_data         );
00353       lua_settable     (pt_state, -3             );
00354    }
00355 
00356    /****************************************/
00357    /****************************************/
00358 
00359    void CLuaUtility::AddToTable(lua_State* pt_state,
00360                                 const std::string& str_key,
00361                                 Real f_data) {
00362       lua_pushstring(pt_state, str_key.c_str());
00363       lua_pushnumber(pt_state, f_data         );
00364       lua_settable  (pt_state, -3             );
00365    }
00366 
00367    /****************************************/
00368    /****************************************/
00369 
00370    void CLuaUtility::AddToTable(lua_State* pt_state,
00371                                 const std::string& str_key,
00372                                 const std::string& str_data){
00373           lua_pushstring(pt_state, str_key.c_str() );
00374           lua_pushstring(pt_state, str_data.c_str());
00375           lua_settable  (pt_state, -3              );
00376    }
00377 
00378    /****************************************/
00379    /****************************************/
00380 
00381    void CLuaUtility::AddToTable(lua_State* pt_state,
00382                                 int n_key,
00383                                 Real f_data) {
00384       lua_pushnumber(pt_state, n_key );
00385       lua_pushnumber(pt_state, f_data);
00386       lua_settable  (pt_state, -3    );
00387    }
00388 
00389    /****************************************/
00390    /****************************************/
00391 
00392    void CLuaUtility::AddToTable(lua_State* pt_state,
00393                                 const std::string& str_key,
00394                                 const CRadians& c_data) {
00395       lua_pushstring(pt_state, str_key.c_str()  );
00396       lua_pushnumber(pt_state, c_data.GetValue());
00397       lua_settable  (pt_state, -3               );
00398    }
00399 
00400    /****************************************/
00401    /****************************************/
00402 
00403    void CLuaUtility::AddToTable(lua_State* pt_state,
00404                                 int n_key,
00405                                 const CRadians& c_data) {
00406       lua_pushnumber(pt_state, n_key            );
00407       lua_pushnumber(pt_state, c_data.GetValue());
00408       lua_settable  (pt_state, -3               );
00409    }
00410 
00411    /****************************************/
00412    /****************************************/
00413 
00414    void CLuaUtility::AddToTable(lua_State* pt_state,
00415                                 const std::string& str_key,
00416                                 const CVector2& c_data) {
00417       StartTable(pt_state, str_key               );
00418       AddToTable(pt_state, "_type", TYPE_VECTOR2 );
00419       AddToTable(pt_state, "x",     c_data.GetX());
00420       AddToTable(pt_state, "y",     c_data.GetY());
00421       EndTable  (pt_state                        );
00422    }
00423    
00424    /****************************************/
00425    /****************************************/
00426 
00427    void CLuaUtility::AddToTable(lua_State* pt_state,
00428                                 int n_key,
00429                                 const CVector2& c_data) {
00430       StartTable(pt_state, n_key                 );
00431       AddToTable(pt_state, "_type", TYPE_VECTOR2 );
00432       AddToTable(pt_state, "x",     c_data.GetX());
00433       AddToTable(pt_state, "y",     c_data.GetY());
00434       EndTable  (pt_state                        );
00435    }
00436    
00437    /****************************************/
00438    /****************************************/
00439 
00440    void CLuaUtility::AddToTable(lua_State* pt_state,
00441                                 const std::string& str_key,
00442                                 const CVector3& c_data) {
00443       StartTable(pt_state, str_key               );
00444       AddToTable(pt_state, "_type", TYPE_VECTOR3 );
00445       AddToTable(pt_state, "x",     c_data.GetX());
00446       AddToTable(pt_state, "y",     c_data.GetY());
00447       AddToTable(pt_state, "z",     c_data.GetZ());
00448       EndTable  (pt_state                        );
00449    }
00450    
00451    /****************************************/
00452    /****************************************/
00453 
00454    void CLuaUtility::AddToTable(lua_State* pt_state,
00455                                 int n_key,
00456                                 const CVector3& c_data) {
00457       StartTable(pt_state, n_key                 );
00458       AddToTable(pt_state, "_type", TYPE_VECTOR3 );
00459       AddToTable(pt_state, "x",     c_data.GetX());
00460       AddToTable(pt_state, "y",     c_data.GetY());
00461       AddToTable(pt_state, "z",     c_data.GetZ());
00462       EndTable  (pt_state                        );
00463    }
00464    
00465    /****************************************/
00466    /****************************************/
00467 
00468    void CLuaUtility::AddToTable(lua_State* pt_state,
00469                                 const std::string& str_key,
00470                                 const CQuaternion& c_data) {
00471       CRadians cAngle;
00472       CVector3 cAxis;
00473       c_data.ToAngleAxis(cAngle, cAxis);
00474       StartTable(pt_state, str_key                 );
00475       AddToTable(pt_state, "_type", TYPE_QUATERNION);
00476       AddToTable(pt_state, "angle", cAngle         );
00477       AddToTable(pt_state, "axis",  cAxis          );
00478       EndTable  (pt_state                          );
00479    }
00480    
00481    /****************************************/
00482    /****************************************/
00483 
00484    void CLuaUtility::AddToTable(lua_State* pt_state,
00485                                 int n_key,
00486                                 const CQuaternion& c_data) {
00487       CRadians cAngle;
00488       CVector3 cAxis;
00489       c_data.ToAngleAxis(cAngle, cAxis);
00490       StartTable(pt_state, n_key                   );
00491       AddToTable(pt_state, "_type", TYPE_QUATERNION);
00492       AddToTable(pt_state, "angle", cAngle         );
00493       AddToTable(pt_state, "axis",  cAxis          );
00494       EndTable  (pt_state                          );
00495    }
00496    
00497    /****************************************/
00498    /****************************************/
00499 
00500    void CLuaUtility::AddToTable(lua_State* pt_state,
00501                                 const std::string& str_key,
00502                                 const CColor& c_data) {
00503       StartTable(pt_state, str_key                   );
00504       AddToTable(pt_state, "_type", TYPE_COLOR       );
00505       AddToTable(pt_state, "red",   c_data.GetRed()  );
00506       AddToTable(pt_state, "green", c_data.GetGreen());
00507       AddToTable(pt_state, "blue",  c_data.GetBlue() );
00508       EndTable  (pt_state                            );
00509    }
00510    
00511    /****************************************/
00512    /****************************************/
00513 
00514    void CLuaUtility::AddToTable(lua_State* pt_state,
00515                                 int n_key,
00516                                 const CColor& c_data) {
00517       StartTable(pt_state, n_key                     );
00518       AddToTable(pt_state, "_type", TYPE_COLOR       );
00519       AddToTable(pt_state, "red",   c_data.GetRed()  );
00520       AddToTable(pt_state, "green", c_data.GetGreen());
00521       AddToTable(pt_state, "blue",  c_data.GetBlue() );
00522       EndTable  (pt_state                            );
00523    }
00524    
00525    /****************************************/
00526    /****************************************/
00527 
00528    int CLuaUtility::LOGWrapper(lua_State* pt_state) {
00529       return LoggerWrapper(LOG, pt_state);
00530    }
00531       
00532    /****************************************/
00533    /****************************************/
00534 
00535    int CLuaUtility::LOGERRWrapper(lua_State* pt_state) {
00536       return LoggerWrapper(LOGERR, pt_state);
00537    }
00538 
00539    /****************************************/
00540    /****************************************/
00541 
00542    int CLuaUtility::LoggerWrapper(CARGoSLog& c_log,
00543                                   lua_State* pt_state) {
00544       /* Get number of arguments */
00545       UInt32 unArgc = lua_gettop(pt_state);
00546       /* Send arguments to log one by one */
00547       UInt32 unType;
00548       for(UInt32 i = 1; i <= unArgc; ++i) {
00549          unType = lua_type(pt_state, i);
00550          switch(unType) {
00551             case LUA_TBOOLEAN: c_log << lua_toboolean(pt_state, i); break;
00552             case LUA_TNUMBER:  c_log << lua_tonumber (pt_state, i); break;
00553             case LUA_TSTRING:  c_log << lua_tostring (pt_state, i); break;
00554             default: c_log << lua_typename (pt_state, unType); break;
00555          }
00556       }
00557       c_log << std::endl;
00558       /* No result is calculated */
00559       return 0;
00560    }
00561 
00562    /****************************************/
00563    /****************************************/
00564 
00565 }
```

---

Generated on 10 Jul 2018 for ARGoS by 
 1.6.1
